# Waning Antibody Responses in Asymptomatic and Symptomatic SARS-CoV-2 Infection

**DOI:** 10.3201/eid2701.203515

**Published:** 2021-01

**Authors:** Pyoeng Gyun Choe, Chang Kyung Kang, Hyeon Jeong Suh, Jongtak Jung, Kyoung-Ho Song, Ji Hwan Bang, Eu Suk Kim, Hong Bin Kim, Sang Won Park, Nam Joong Kim, Wan Beom Park, Myoung-don Oh

**Affiliations:** Seoul National University College of Medicine, Seoul, South Korea

**Keywords:** waning immunity, neutralizing antibodies, ELISA, COVID-19, SARS-CoV-2, respiratory infections, severe acute respiratory syndrome coronavirus 2, 2019 novel coronavirus disease, coronavirus disease, zoonoses, viruses, coronavirus, antibodies

## Abstract

We investigated the kinetics of severe acute respiratory syndrome coronavirus 2 neutralizing antibodies in 7 asymptomatic persons and 11 patients with pneumonia. The geometric mean titer of neutralizing antibodies declined from 219.4 at 2 months to 143.7 at 5 months after infection, indicating a waning antibody response.

Neutralizing antibodies develop in asymptomatic persons with severe acute respiratory syndrome coronavirus 2 (SARS-CoV-2) infection; however, the initial immune response is not as strong as in patients with more severe disease (*1*,*2*). We investigated the kinetics of SARS-CoV-2 neutralizing antibodies during the 5 months after infection in asymptomatic persons and patients with pneumonia caused by SARS-CoV-2.

We studied 7 persons infected with SARS-CoV-2 who were isolated in a community treatment center operated by Seoul National University (SNU) Hospital in Daegu, South Korea (*3*). Comprehensive monitoring confirmed that these 7 patients were asymptomatic (*4*). We also evaluated 11 SARS-CoV-2–positive patients with pneumonia at the Biocontainment Unit in the SNU Hospital and SNU Bundang Hospital. We classified each case of pneumonia as subtle (i.e., infiltrations observed only on computed tomography) or apparent (i.e., infiltrations observed on plain chest radiograph) (Appendix). All patients provided informed consent.

We evaluated the antibody responses at 2 and 5 months after infection, as reported (*1*). We semiquantitatively measured IgG against SARS-CoV-2 using ELISA (Euroimmun, https://www.euroimmun.com) with the recombinant S1 domain of the SARS-CoV-2 spike protein as the antigen. We interpreted the optical density ratio (sample/calibrator) as negative (<0.8), borderline (>0.8 to <1.1), or positive (>1.1), according to the manufacturer’s recommendations. We also conducted neutralization assays as previously described (*5*) using BetaCoV/Korea/SNU01/2020 virus (*6*) and 2-fold serially diluted plasma samples (2–4,096-fold). We recorded the highest dilution of plasma that showed inhibition activity of SARS-CoV-2 as the neutralizing antibody titer. We considered a >4-fold reduction in antibody titer to be a waning response. The Institutional Review Boards of Seoul National University Hospital approved the study (IRB no. H-2004-158-1118).

Two months after infection, 11 (100%) patients with pneumonia and 5 (71%) with asymptomatic infection had positive ELISA results. Five months after infection, 5 (100.0%) patients with apparent pneumonia, 5 (83.3%) with subtle pneumonia, and 4 (57.1%) with asymptomatic infection had positive ELISA results. The mean ELISA optical density decreased significantly from 2 to 5 months after infection (4.93 at 2 months vs. 4.09 at 5 months; p = 0.01).

Two months after infection, all patients had neutralizing antibodies. Antibody titers correlated with disease severity; the geometric mean titer was 105 among symptomatic persons, 161 among patients with subtle pneumonia, and 891 among patients with apparent pneumonia. Five months after infection, all patients still had neutralizing antibodies, but the geometric mean titer decreased significantly (219.4 at 2 months vs. 143.7 at 5 months; p = 0.03). In the linear regression model, the decline was significantly associated with the antibody levels at 2 months as measured by ELISA (r = 0.536, p = 0.02) and the neutralization assay (r = 0.563, p = 0.02) (Appendix Figure). The waning neutralizing antibody response occurred in 2 (40%) of 5 patients with apparent pneumonia and 2 (33%) of 6 with subtle pneumonia, but none of the asymptomatic persons (Figure).

**Figure:**
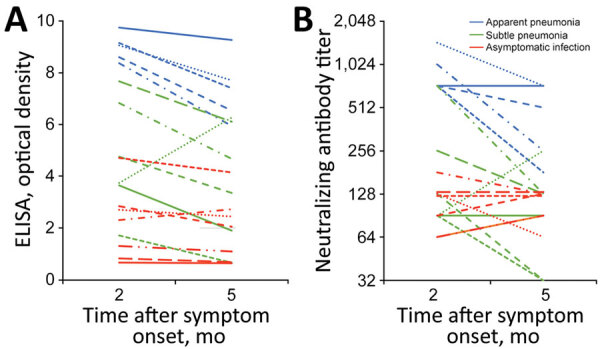
Waning antibody response against severe acute respiratory virus coronavirus 2, South Korea, 2020. Responses measured by A) ELISA optical density measurements (p = 0.01); B) neutralizing antibody titers (p = 0.03). Each line indicates data from a single patient.

Determining the longevity of humoral immunity to SARS-CoV-2 is essential to predicting herd immunity to coronavirus disease. Among patients with severe acute respiratory syndrome coronavirus, which is closely related to SARS-CoV-2, a total of 90% maintained IgG for 2 years and 50% for 3 years (*7*). However, humoral immunity to common human coronavirus is short-lived; antibodies against seasonal coronaviruses return to baseline levels by 52 weeks after infection, enabling homologous reinfections (*8*). A recent study showed that the antibody titers of patients with mild coronavirus disease declined more quickly than did those of patients with severe acute respiratory syndrome (*9*).

Our findings demonstrate waning humoral immunity in patients with SARS-CoV-2 infection. We documented the decline of neutralizing antibody titers in asymptomatic and symptomatic patients. In this study, the initial neutralizing antibody reaction appeared to correlate with the severity of the disease. However, patients with pneumonia were considerably older than asymptomatic persons, and increasing age is associated with a stronger neutralizing antibody response (*10*). In this study, neutralizing antibody titer decreased more in symptomatic than asymptomatic patients. Our study reinforces the concern that naturally acquired humoral immunity against SARS-CoV-2 might not be long-lasting.

Appendix Further information on characteristics of study participants and association between baseline antibody levels and waning antibody response in persons with asymptomatic and symptomatic severe acute respiratory syndrome coronavirus 2 infection.
