# Supplementary material for: Waning Antibody Responses in Asymptomatic and Symptomatic SARS-CoV-2 Infection
Source: Emerg Infect Dis. 2021 Jan;27(1):327–9. doi: 10.3201/eid2701.203515 (PMC7774548; doi:10.3201/eid2701.203515)
Supplement: Appendix — Further information on characteristics of study participants and association between baseline antibody levels and waning antibody response in persons with asymptomatic and symptomatic severe acute respiratory syndrome coronavirus 2 infection. [file 20-3515-Techapp-s1.pdf]

Article DOI: https://doi.org/10.3201/eid2701.203515

Waning Antibody Responses in Asymptomatic and Symptomatic SARS-CoV-2 Infection

Appendix

Appendix Table. Clinical characteristics of patients with severe acute respiratory syndrome coronavirus 2, South Korea, 2020.

	Severity, no. (%)*		
Characteristics	Completely asymptomatic	Subtle pneumonia	Apparent pneumonia
Total	7	6	5
M	5 (71.4)	2 (33.3)	4 (80.0)
F	2 (28.6)	4 (66.7)	1 (20.0)
Age, median years (range)	25 (20–28)	47 (24–60)	48 (39–69)
Concurrent conditions			
Hypertension	0	0	2 (40.0)
Diabetes mellitus	0	2 (33.3)	1 (20.0)
Initial lymphocyte counts (cells/µL), median (range)	NA	1,170 (789–2,051)	1,028 (652–1,780)
Initial C-reactive protein (mg/dL), median (range)	NA	0.21 (0.08–1.32)	5.09 (3.89–7.28)
On oxygen therapy	0	0	1 (20.0)
Antiviral treatment (lopinavir/ritonavir)	0	1 (16.7)	4 (80.0)
ELISA results at 2 mo after infection			
Negative	1 (14.3)	0	0
Borderline	1 (14.3)	0	0
Positive	5 (71.4)	6 (100.0)	5 (100.0)
ELISA results at 5 mo after infection			
Negative	2 (28.6)	1 (16.7)	0
Borderline	1 (14.3)	0	0
Positive	4 (57.1)	5 (83.3)	5 (100.0)

*Unless otherwise indicated

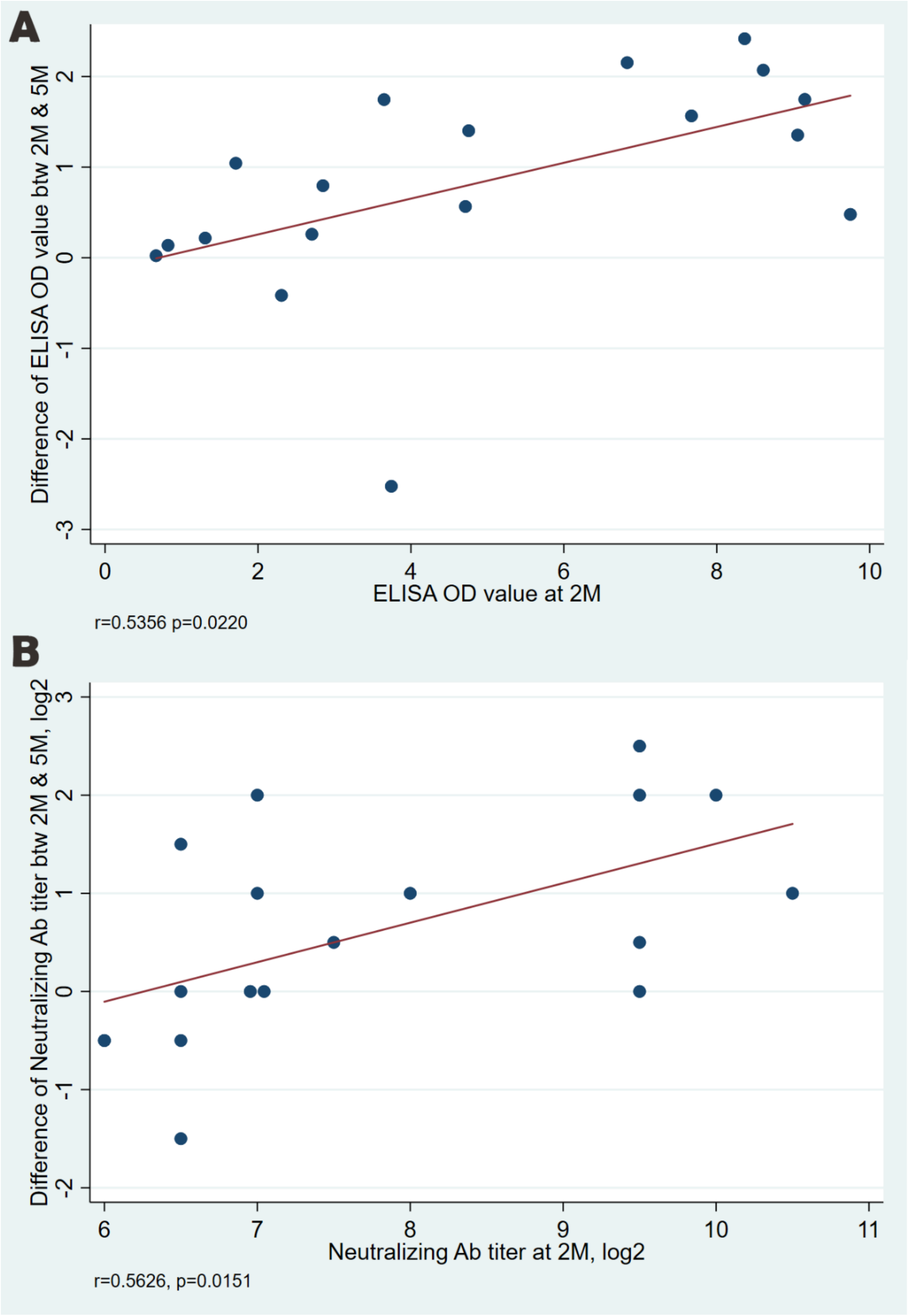

Appendix Figure. Association between baseline levels and total decline of antibodies against severe acute respiratory syndrome coronavirus 2, South Korea, 2020. Measured by A) ELISA optical density values and B) neutralizing antibody titers.
